# Ultra-widefield optical coherence tomography angiography in diabetic retinopathy: from retinal lesions to choroidal metrics

**DOI:** 10.3389/fmed.2026.1885710

**Published:** 2026-06-22

**Authors:** Tianhong Luo, Yingshi Zou, Yali Gao

**Affiliations:** 1 Department of Ophthalmology, Shenzhen People’s Hospital, The Second Clinical Medical College Jinan University, Shenzhen, Guangdong, China; 2 Department of Ophthalmology, Shenzhen People’s Hospital (The First Affiliated Hospital, Southern University of Science and Technology, The Second Clinical Medical College, Jinan University), Shenzhen, Guangdong, China

**Keywords:** choroidal metrics, diabetic lesions, diabetic retinopathy, ultra-widefield optical coherence tomography angiography, vitreoretinal interface

## Abstract

Diabetic retinopathy (DR) continues to represent one of the principal etiologies of visual impairment among working-age populations, and the escalating global burden of diabetes mellitus suggests that this challenge will likely intensify in the coming decades. Conventional diagnostic modalities, most notably fluorescein angiography (FA), have long been employed as reference standards in clinical practice; nevertheless, these approaches are hampered by inherent drawbacks, including their invasive nature and the absence of depth-resolved structural information. The advent of optical coherence tomography angiography (OCTA) has provided a compelling non-invasive alternative capable of delivering depth-stratified vascular visualization. Subsequent technological refinements have yielded ultra-widefield (UWF) OCTA, which substantially extends the imaging field of view and affords detailed examination of the peripheral retinal vasculature that previously remained beyond the reach of conventional acquisition systems. The present review synthesizes current evidence regarding the clinical utility of UWF-OCTA in DR, with particular emphasis on its capacity to identify and characterize key retinal microvascular lesions, encompassing neovascularization (NV), non-perfusion areas (NPAs), microaneurysms (MAs), and intraretinal microvascular abnormalities (IRMAs). The vitreoretinal interface (VRI) slab for detecting NV, emerging choroidal metrics, and a comparison between UWF-OCTA and UWF-FA are also discussed. The evidence suggests that UWF-OCTA detects NV with high sensitivity and specificity, compares favorably to FA, and offers unique advantages, although limitations such as segmentation errors and a smaller field of view must be acknowledged. UWF-OCTA appears to be a promising non-invasive tool for DR diagnosis and monitoring, potentially complementing rather than completely replacing conventional angiography.

## Graphical abstract

**Figure:**
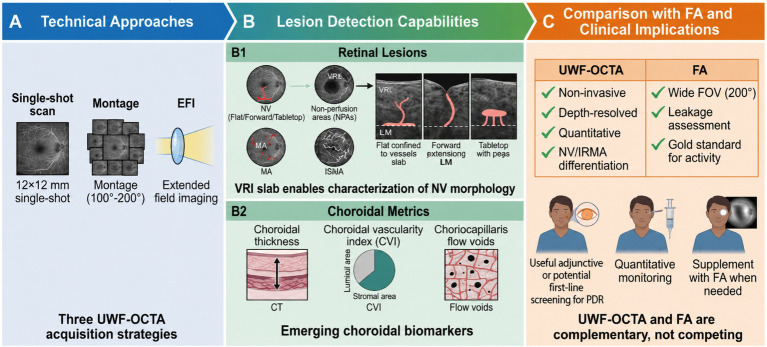

## 1 Introduction

Diabetes mellitus has emerged as a substantial public health challenge of global magnitude, with the worldwide prevalence of this condition projected to escalate from approximately 8.8% in 2015 to an estimated 10.4% by 2040 (1). Diabetic retinopathy (DR), recognized as among the most prevalent microvascular sequelae of diabetes, affects roughly one-third of all individuals living with the disease and persists as a foremost contributor to irreversible blindness in the adult population, notwithstanding considerable progress in both diagnostic capabilities and therapeutic interventions (2–5). The visual morbidity attributable to DR carries disproportionate consequences for individuals of working age, imposing far-reaching socioeconomic ramifications on affected patients and placing a measurable strain on healthcare systems at large (2, 6).

The categorization and severity stratification of DR have historically depended upon stereoscopic color fundus photography, with the Early Treatment Diabetic Retinopathy Study (ETDRS) grading system, a 13-level hierarchical scale derived from seven-field photographic acquisition, having functioned as the accepted gold standard over many years (7). Fluorescein angiography (FA) has similarly been extensively adopted as a complementary diagnostic instrument, given its capacity to reveal blood-retinal barrier disruption, microaneurysms (MAs), non-perfusion areas (NPAs), intraretinal microvascular abnormalities (IRMAs), and neovascularization (NV) (8). Nevertheless, FA has several clinically relevant limitations. It is invasive, requires intravenous dye administration, and carries a small but non-negligible risk of adverse reactions. Its use may also be restricted in patients with renal insufficiency or pregnancy. In addition, FA provides two-dimensional projection images, lacks independent depth-resolved visualization of retinal vascular plexuses, and remains predominantly qualitative for disease assessment (8).

The introduction of optical coherence tomography angiography (OCTA) represented a turning point in the management of DR (9–11). OCTA represents a derivative of optical coherence tomography that produces high-resolution angiographic images through the detection of motion contrast generated by circulating erythrocytes across sequentially acquired B-scans. Contemporary OCTA platforms operate by capturing clusters of two to four B-scans along the x fast axis at successive points on the y slow scan axis, whereupon dedicated software algorithms isolate and reconstruct the flow signal from each acquired cluster (9). In comparison with conventional dye-based angiographic techniques, OCTA confers several notable advantages, most prominently the complete elimination of exogenous contrast agent administration alongside the provision of depth-stratified vascular information encompassing both the superficial and deep retinal capillary networks. The spatial resolution achievable with OCTA substantially exceeds that attainable through FA, and the modality additionally facilitates quantitative image analysis, enabling systematic measurement of parameters including vessel density, vessel length density, perfusion density, as well as the dimensions and morphological configuration of the foveal avascular zone (12).

Over an extended period, the imaging coverage of OCTA remained constrained to acquisitions of approximately 6 × 6 mm, representing a substantial limitation in peripheral retinal evaluation. Subsequent investigative endeavors aimed at broadening this restricted field of view have yielded progressively encouraging outcomes in recent years (13, 14). The emergence of widefield and ultra-widefield OCTA has heralded an advancement in the clinical assessment and management of retinal vascular disorders, DR included. These innovative imaging platforms confer diagnostic benefits, particularly with respect to the identification of peripheral non-perfusion areas (NPAs) and neovascularization (NV), pathological features that are frequently situated beyond the boundaries of the central macular region and thus remain inaccessible to conventional narrow-field acquisition strategies (15, 16).

**Figure 1:**
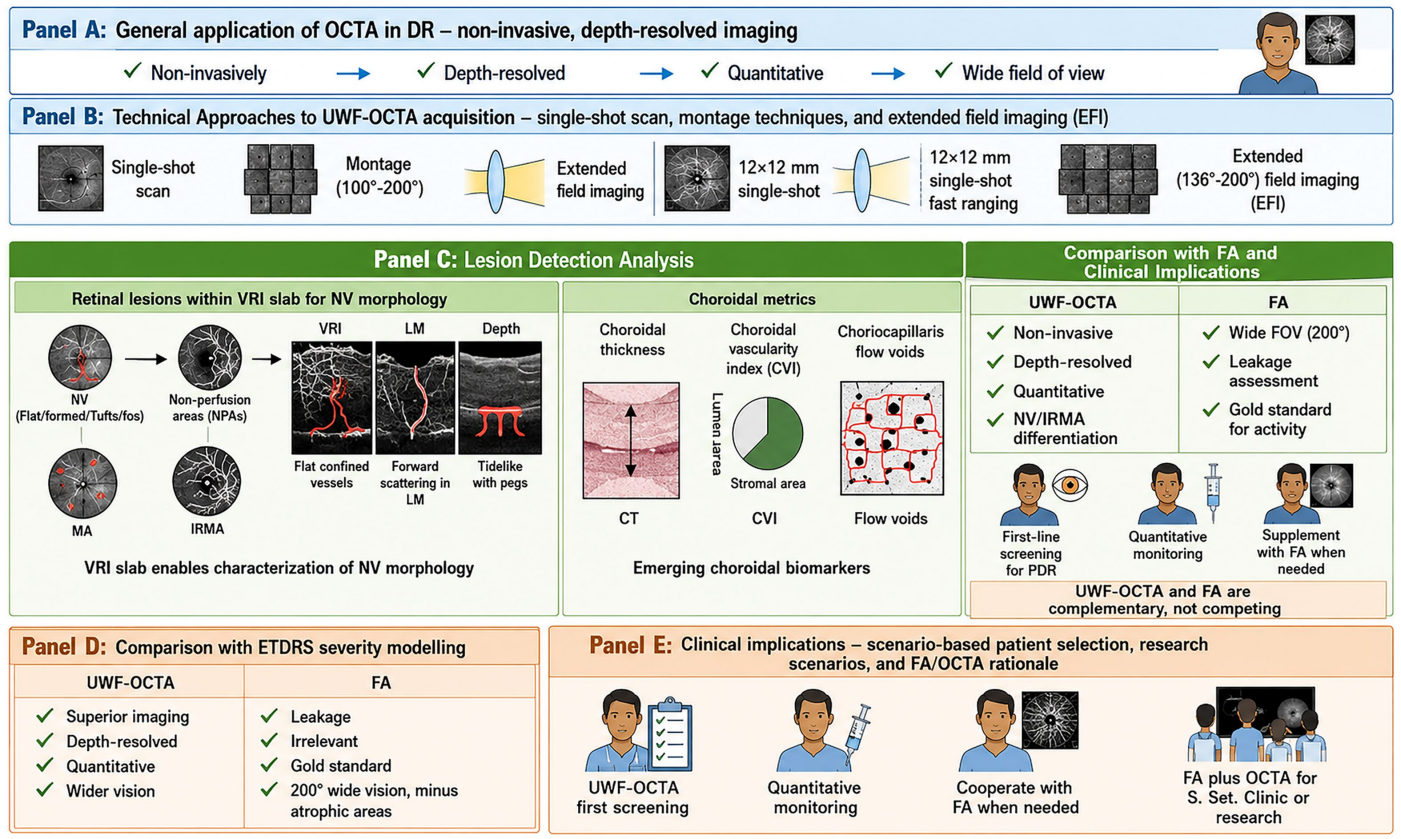
Schematic diagram illustrating the logical structure of the review. **(A)** General applications of OCTA in diabetic retinopathy, including non-invasive, depth-resolved, quantitative, and widefield imaging. **(B)** Technical approaches to UWF-OCTA acquisition, including single-shot scanning, montage techniques, and extended field imaging. **(C)** Detection and characterization of retinal lesions and emerging choroidal metrics. **(D)** Comparison with ETDRS between UWF-OCTA and fluorescein angiography. **(E)** Clinical implications, scenario-based patient selection, research priorities, and the complementary roles of UWF-OCTA and fluorescein angiography.

The current review is designed to furnish a thorough synthesis of the clinical and investigational role of UWF-OCTA in the context of DR, organized according to a coherent and systematically structured logical framework that is schematically represented in Figure 1. As depicted therein, the discourse commences with a foundational examination of the broader applicability of OCTA in DR management, with particular attention directed toward its inherent non-invasive character and depth-stratified imaging capabilities, attributes that collectively confer distinct diagnostic superiority over established conventional imaging approaches. The review then moves to a detailed examination of the technical approaches that enable UWF imaging. Specifically, single-shot widefield scans, montage techniques, and extended field imaging (EFI) and considers how different acquisition methods influence subsequent image quality and diagnostic performance. From there, the figure depicts a multi-level analysis of retinal and choroidal features: at the retinal level, the focus is on the detection and characterization of key diabetic lesions, including NV, NPAs, MAs, and IRMAs, with particular attention to the vitreoretinal interface (VRI) slab for detailed morphological classification of NV; at the choroidal level, emerging metrics such as choroidal thickness, choroidal vascularity index (CVI), and choriocapillaris flow voids are discussed as potential complementary biomarkers. The figure then transitions to a comparative analysis between UWF-OCTA and FA, evaluating their respective strengths and limitations in terms of lesion detection, leakage assessment, quantitative metrics, and practical clinical utility. Finally, as shown in the Figure 1. the review concludes by synthesizing this evidence into scenario-based clinical recommendations, identifying the current best-application scenarios for UWF-OCTA, the situations where FA remains necessary, and the key research bottlenecks that still need to be addressed.

Building upon this contextual foundation, the present article endeavors to consolidate and critically appraise the existing body of evidence with the aim of elucidating the practical diagnostic and therapeutic value that UWF-OCTA holds within the broader framework of DR clinical management.

## 2 Methods

A focused structured literature search was conducted to support this narrative review. PubMed and Web of Science were searched for English-language publications from January 2000 to March 2026. The search strategy was revised to include both abbreviations and corresponding full terms related to diabetic retinopathy, widefield or ultra-widefield OCTA, retinal lesions, and choroidal indicators. Because this article was designed as a structured narrative review rather than a systematic review or meta-analysis, the search was intended to identify representative and clinically relevant literature rather than to exhaustively retrieve and quantitatively synthesize all available studies.

The core PubMed search strategy was: (“DR”[Title/Abstract] OR “diabetic retinopathy”[Title/Abstract] OR “Diabetic Retinopathy”[MeSH Terms]) AND (“UWF-OCTA”[Title/Abstract] OR “WF-OCTA”[Title/Abstract] OR “ultra-widefield OCTA”[Title/Abstract] OR “widefield OCTA”[Title/Abstract] OR “ultra-widefield optical coherence tomography angiography”[Title/Abstract] OR “widefield optical coherence tomography angiography”[Title/Abstract] OR (“OCTA”[Title/Abstract] AND (“widefield”[Title/Abstract] OR “ultra-widefield”[Title/Abstract] OR montage[Title/Abstract] OR “extended field imaging”[Title/Abstract] OR EFI[Title/Abstract]))) AND (“NV”[Title/Abstract] OR “neovascularization”[Title/Abstract] OR “neovascularisation”[Title/Abstract] OR “non-perfusion”[Title/Abstract] OR nonperfusion[Title/Abstract] OR “non-perfusion areas”[Title/Abstract] OR “nonperfusion areas”[Title/Abstract] OR “MAs”[Title/Abstract] OR microaneurysm[Title/Abstract] OR microaneurysms[Title/Abstract] OR “IRMAs”[Title/Abstract] OR IRMA[Title/Abstract] OR “intraretinal microvascular abnormalities”[Title/Abstract] OR “VRI”[Title/Abstract] OR “vitreoretinal interface”[Title/Abstract] OR “choroidal thickness”[Title/Abstract] OR “CVI”[Title/Abstract] OR “choroidal vascularity index”[Title/Abstract] OR “choriocapillaris flow voids”[Title/Abstract] OR “choriocapillaris flow deficits”[Title/Abstract]). The Web of Science strategy used the same conceptual term groups adapted to topic-field searching.

**Figure 2:**
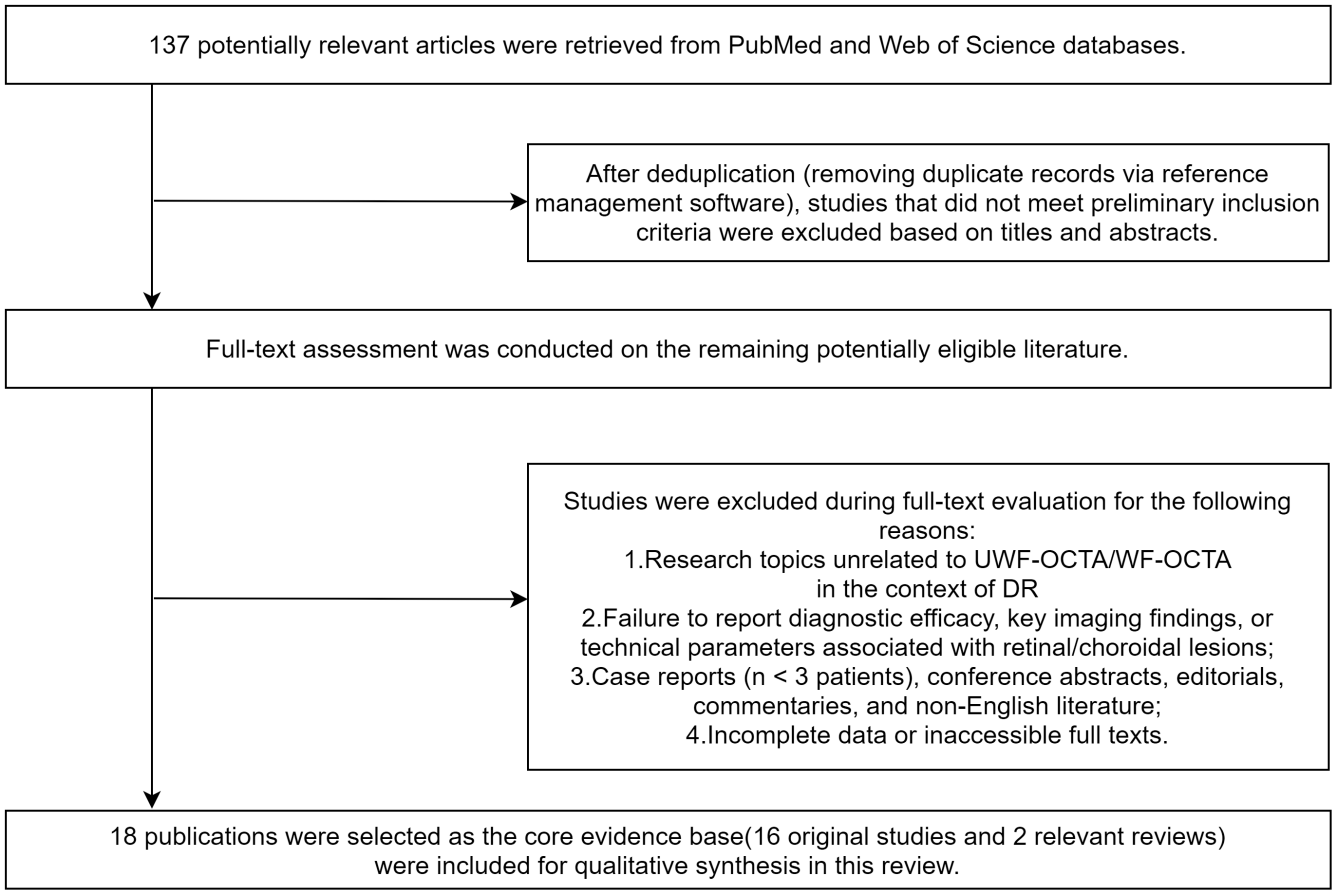
Flowchart for literature retrieval and screening: from this process, a total of 18 articles were deemed relevant and were included in this review.

The preliminary search retrieved approximately 137 potentially relevant articles. Following the removal of duplicate records, the titles and abstracts of all remaining publications were independently evaluated to assess their suitability for inclusion. Eligibility criteria were defined as follows: (1) original investigational or review articles appearing in peer-reviewed journals publishing exclusively in the English language; (2) studies examining UWF-OCTA or widefield OCTA (WF-OCTA) within the clinical or investigational framework of DR; (3) articles reporting outcomes pertaining to diagnostic efficacy, characteristic imaging manifestations, or technically relevant parameters associated with retinal or choroidal pathology. Publications classified as conference abstracts, case series encompassing fewer than three patients, editorials, and articles composed in languages other than English were systematically excluded from consideration. Among the references cited in this review, 18 were identified from the focused database search, including 16 original studies and 2 review articles. Additional references were included through manual screening of reference lists and targeted supplementary searches to provide methodological, historical, and background context, including foundational literature on DR classification, OCTA principles, fluorescein angiography, imaging artifacts, segmentation methods, choroidal metrics, and artificial intelligence. The literature retrieval and selection process is summarized in Figure 2.

## 3 Technical approaches to achieving UWF-OCTA

The acquisition of UWF-OCTA images can be achieved through several approaches, mainly extended field imaging (EFI), montage techniques, and single-shot widefield scans, each of which differs in field of view, resolution, acquisition time, and susceptibility to artifacts (13, 17). Before discussing these approaches, it is important to clarify the terminology used in this review, because the terms “widefield,” “ultra-widefield,” “montage,” and “single-shot” are not always used consistently across studies.

### 3.1 WF-OCTA

This term generally refers to OCTA imaging with a field of view larger than conventional 6 × 6 mm macular scans but usually less than approximately 60°, corresponding to 12 × 12 mm or 15 × 9 mm acquisitions on most commercial devices. Single-shot WF-OCTA can be obtained without image stitching and is useful for evaluating the posterior pole and perimacular retina, but it may not fully capture mid-peripheral or far-peripheral lesions.

### 3.2 UWF-OCTA

This term is typically reserved for imaging that covers 100° or more of the retina. It is almost always achieved through montage techniques (see below), as current single-shot technology does not reliably achieve such fields without substantial resolution loss or artifacts. From a conceptual standpoint, UWF-OCTA bears considerable resemblance to UWF-FA with regard to the extent of retinal territory it is designed to encompass; however, the effective field of view achievable in practical clinical implementations consistently falls short of that attainable through UWF-FA across the majority of currently available acquisition systems.

### 3.3 Montage OCTA

This technique involves acquiring multiple individual OCTA volumes (usually 3 × 3 mm, 6 × 6 mm, or 12 × 12 mm) at different fixation positions and subsequently stitching them together using software algorithms. Montage imaging can achieve fields of view up to 100°–200°, depending on the number of constituent scans. However, it is more time-consuming, requires active patient cooperation, and is susceptible to registration errors, motion artifacts, and illumination inhomogeneity at stitch boundaries. The quality of montage images also varies considerably across different commercial platforms.

### 3.4 Single-shot WF-OCTA

This refers to acquisition of a single OCTA volume with a wide field of view (usually 12 × 12 mm or 15 × 9 mm) without image stitching. It is faster and less prone to motion artifacts than montage but is limited to approximately 40°–60° of retinal coverage. Some newer devices claim single-shot fields approaching 80°, but independent validation is still limited.

### 3.5 EFI

This approach constitutes an optical methodology in which a positive diopter lens is strategically positioned between the OCTA probe and the ocular surface to expand the incident light angle and increase the effective field of view. It can be applied to either single-shot or montage acquisitions, but this wider coverage is obtained at the cost of reduced lateral resolution, which may affect the detection of small lesions such as MAs.

A clear understanding of these distinctions is essential for interpreting the comparative diagnostic performance data discussed in later sections, as the reported sensitivity and specificity for detecting specific lesions such as NV and NPAs are, to a certain extent, dependent on whether single-shot or montage UWF-OCTA was used and on the actual field of view achieved.

### 3.6 Extended field imaging

EFI expands the OCTA field of view by placing a positive diopter lens between the imaging probe and the ocular surface, thereby increasing the angle of incident light (17). Uji and Yoshimura (17) compared conventional spectral-domain and swept-source OCT acquisitions with EFI-assisted acquisitions on the same systems and found significant increases in both horizontal and vertical scan dimensions. When combined with swept-source OCT, EFI produced an effective angular field of approximately 60°–70°, indicating that this method can expand coverage without requiring structural modification of the OCT device (17).

When the EFI technique was applied to Swept-Source Optical Coherence Tomography Angiography (SS-OCTA), it resulted in a larger scan size than both SS-OCTA images acquired without EFI and traditional FA captured using a 55° lens (13). In eyes with DR, EFI-SS-OCTA showed sensitivity of 96 and 79%, and specificity of 100 and 96%, for detecting NPAs and NV, respectively, compared with FA (13). In a parallel investigational framework, Pellegrini and colleagues undertook a systematic assessment of the spatial extent of NPAs alongside the prevalence and enumeration of NV lesions (18). Beyond demonstrating a substantially broader captured fundus area, EFI-SS-OCTA additionally disclosed a considerably greater territorial extension of NPAs relative to both non-EFI SS-OCTA and FA, a finding that underscores the incremental diagnostic yield conferred by the EFI approach in peripheral retinal evaluation.

However, EFI has important limitations. Because the same number of A-scans is distributed across a larger retinal area, image enlargement is accompanied by reduced sampling density and lower slab resolution. This may influence quantitative measurements by underestimating vessel density or overestimating NPA extent. Therefore, findings derived from EFI should be interpreted cautiously, particularly when small lesions or quantitative perfusion metrics are being evaluated.

### 3.7 Montage technique

The montage methodology expands the imaging field by combining multiple smaller scans into a single composite image (14, 19–21). Considerable heterogeneity exists among the montage protocols documented across the published literature, and the absence of rigorous methodological standardization governing these acquisition strategies constitutes a plausible source of inter-study variability in reported imaging outcomes and diagnostic performance metrics.

de Carlo et al. (19) were among the early investigators to apply montage OCTA for wider retinal vascular visualization. In a small prospective case series, nine adjacent 3 × 3 mm OCTA scans were combined to create an approximately 8 × 8 mm widefield montage image, corresponding to a 30° field of view. This montage provided better visualization of retinal vascular architecture than a single 8 × 8 mm OCTA scan and detected more retinal pathology than either FA alone or single-scan OCTA when compared with 50° FA (19).

In a more recent investigational effort, Lavia et al. (20) later used approximately 25 individual 3 × 3 mm OCTA scans extending from the fovea toward the periphery to generate widefield images in healthy subjects (13). This method reduced low-signal artifacts associated with larger scans and improved layer segmentation and quantitative reliability (20).

In eyes with PDR, Zhang et al. (14) reconstructed a 100° field of view by integrating sixteen 6 × 6 mm scans. The resulting montage WF-OCTA images provided detailed visualization of retinal vascular plexuses and revealed a larger burden of peripheral microvascular pathology than conventional FA (12). These findings suggest that montage OCTA may be particularly useful for assessing peripheral retinal vascular abnormalities in DR.

The main disadvantage of montage acquisition is its susceptibility to artifacts. These include projection artifacts, signal attenuation, segmentation errors, vessel duplication, spatial misregistration, blink artifacts, motion distortion, and discontinuities at stitch boundaries (21). Image quality may be especially compromised in eyes with advanced DR, NV, epiretinal membranes, DME, pigment epithelial detachment, or media opacity (21). These factors should be considered when interpreting montage OCTA images, particularly in studies reporting lesion detection rates or quantitative vascular parameters.

### 3.8 Single-shot scans

Newer SS-OCTA systems can acquire single-shot WF images with scan dimensions of 15 × 9 mm or 12 × 12 mm, corresponding to approximately 40° of retinal coverage (22). These individual acquisitions may be subsequently merged to produce larger composite retinal maps extending up to 80° of total retinal coverage (15, 23). Compared with montage approaches, single-shot WF-OCTA is faster, easier to perform, and less dependent on patient fixation. However, the larger scan area is associated with reduced sampling density and lower effective resolution, which may limit visualization of small capillary abnormalities. Hirano et al. (24) compared SS-OCTA scans of different sizes across DR severity levels and found that 3 × 3 mm images had the best diagnostic performance for distinguishing DR eyes from healthy controls. Although this may seem counterintuitive because many DR lesions occur outside the macula, the result may be explained by the higher resolution of smaller scans and the ability to capture subtle early microvascular abnormalities (24, 25). Larger scans may include more macrovascular structures and fewer fine capillary details, potentially reducing sensitivity to early capillary dropout (24).

Accumulating evidence from several investigations has indicated that the earliest detectable microvascular alterations attributable to DR may originate within the perimacular region, a proposition supported by the consistent observation of foveal avascular zone enlargement in affected patients (26). This may partly explain the diagnostic value of small macular-centered scans. In addition, larger 12 × 12 mm and 15 × 9 mm scans are more prone to peripheral distortion and motion artifacts, which can affect both qualitative interpretation and quantitative measurements (27).

From the summary of technical approaches presented above, it becomes apparent that one of the fundamental reasons why different studies often report divergent or even contradictory diagnostic performance data is, to a considerable extent, the substantial heterogeneity in technical methodologies. Several specific aspects deserve particular attention in this regard. First, differences in scan range and montage techniques play a non-negligible role. Second, variations in segmentation algorithms across different OCTA devices (and even across different software versions of the same device) affect the delineation of the internal limiting membrane (ILM) and the retinal pigment epithelium (RPE). Third, lack of uniformity in lesion definitions contributes to cross-study inconsistency. Fourth, differences in the disease spectrum of study populations must be considered.

In summary, when interpreting seemingly conflicting findings across studies, readers need to carefully consider the technical and methodological heterogeneity factors outlined above. This also suggests that future studies should report device parameters, scanning protocols, segmentation algorithms, and lesion interpretation criteria in greater methodological detail to facilitate cross-study comparisons and data integration.

## 4 Detection of retinal lesions using UWF-OCTA

UWF-OCTA features ultra-wide field and depth-resolved advantages, overcoming the view limitation of conventional OCTA and enabling precise detection of DR retinal microvascular lesions. Its clinical application in identifying typical DR retinal lesions is summarized below.

### 4.1 Neovascularization (NV)

#### 4.1.1 Detection using the vitreoretinal interface slab

The vitreoretinal interface (VRI) slab (10 μm to 300 μm above the ILM) is a core technical layer of UWF-OCTA for capturing extraretinal NV stretching into the vitreous cavity, which is critical for identifying neovascularization elsewhere (NVE) and neovascularization of the disc (NVD) in PDR (28–31).

Lu et al. (28) proved the high detection efficiency of VRI-related slabs for NV in a prospective study of 142 diabetic eyes. When SS-OCTA B-scan served as the reference standard, the VRI Angio slab demonstrated a sensitivity of 91.9% for NV identification, a value that appreciably surpassed the 78.6% sensitivity yielded by the VRI Structure slab in isolation. Critically, the concurrent utilization of both slabs in a complementary fashion elevated the overall detection sensitivity to 99.1%. Stratified subgroup analyses further revealed that sensitivity reached 100.0% for neovascularization of the disc (NVD), while neovascularization elsewhere (NVE) was detected with a sensitivity of 90.0%, a figure that could be subsequently augmented to 98.9% through the implementation of combined dual-slab detection protocols (28).

In a morphologically grounded classification framework, Vaz-Pereira et al. (31) stratified neovascular lesions into three distinct subtypes, designated as flat, forward, and tabletop configurations, based upon their characteristic structural and spatial features. Flat NV had a low detection rate (67.9%) on VRI Angio due to ILM segmentation errors, while forward and tabletop NV were nearly fully visualized (98.6, 100.0%); VRI Structure slab could display undetected flat NV as dark inverted signals, and manual ILM segmentation correction realized complete visualization of flat NV (28). Short-term follow-up showed that NV morphological types remained stable (29). The morphology-specific definitions, detection rates, and principal limitations of VRI Angio and VRI Structure imaging are summarized in Table 1.

**Table 1:** Detection rates of NV morphologies on VRI angio and VRI structure slabs.

Morphology	Definition	VRI angio	VRI structure	Combined	Main limitation
Flat NV	Posterior hyaloid face; no ILM breach	67.9% (36/53)	NR	96.2% (51/53)*	ILM segmentation error
Forward NV	Traverses posterior hyaloid into vitreous	98.6% (136/138)	NR	100% (138/138)	Well visualized
Tabletop NV	Anterior displacement by vitreous traction; vascular pegs	100% (43/43)	NR	100% (43/43)	Some segmentation errors possible

*VRI Structure alone was not systematically reported for each morphological type in the original study; the combined detection rate for flat NV (96.2%) reflects the incremental yield from adding VRI Structure to VRI Angio. Manual correction of ILM segmentation allowed visualization of all 17 flat NV initially undetected on VRI Angio.

Overall, the combined VRI Angio and Structure slabs have high sensitivity for NV detection in DR and enable morphological classification, but automated identification of flat NV is limited by segmentation errors, and the cross-device generalizability of detection parameters needs further verification. Thus, VRI slab imaging serves as an auxiliary tool for NV detection, and B-scan correlation or manual segmentation correction is needed to avoid missed diagnosis of flat NV (28).

#### 4.1.2 The VRI: anatomical rationale and clinical importance

The VRI is the anatomical boundary where NV breaks through the retina into the vitreous cavity in PDR, and the VRI slab of OCTA is designed to target this structural feature for capturing extraretinal lesions (32). The 10 μm to 300 μm segmentation range above the ILM can effectively distinguish NV (ILM-breached) from IRMA (intraretinal-confined) (33).

The high sensitivity of VRI Angio slab for NV detection supports its value in PDR screening and disease monitoring (28), while ILM segmentation errors remain the main technical constraint, especially for flat NV; manual correction improves visualization but is time-consuming, and deep learning algorithms may help realize automated detection of flat NV (34). The application of deep learning computational frameworks to DR screening and diagnostic workflows holds considerable promise as a viable strategy for enhancing the automated identification of flat NV lesions that consistently elude reliable detection through conventional segmentation algorithms, thereby offering a potentially transformative means of circumventing the time-consuming and operator-dependent manual correction procedures that currently represent an indispensable yet burdensome component of clinical image analysis (35, 36).

#### 4.1.3 Sensitivity and specificity of UWF-OCTA for NV detection

A substantial and growing body of published evidence has collectively corroborated the diagnostic and clinical management value of WF-OCTA in the context of PDR (37–40), with accumulated data consistently demonstrating that its capacity for NV identification surpasses that achievable through conventional indirect ophthalmoscopy and standard color fundus photography across comparable patient cohorts (38, 39).

In a large-scale investigation, Russell et al. (41–43) documented that WF-OCTA successfully identified NV in 98% of 651 PDR-affected eyes, achieving a sensitivity of 99% among treatment-naive individuals and 97% in those who had previously undergone therapeutic intervention (37). These encouraging performance metrics have been independently corroborated by subsequent investigations employing a range of alternative WF-OCTA montage acquisition strategies (44).

The distribution of NVD and NVE in PDR patients appears to be somewhat heterogeneous. In the study by Russell et al. (41–43), the majority of eyes featured both NVD and NVE (50%), while 40% displayed NVE only, and 10% had NVD only (37). NVE was mainly distributed in the superotemporal quadrants, and treatment history did not affect the distribution of NV (37). A systematic review showed that WF-OCTA had high sensitivity (96.55%) and specificity (94.74%) for NV detection, which was comparable to WF-FA, and the two modalities were complementary (45). The capacity of WF-OCTA to reliably identify peripheral NVE lesions may be constrained by the inherent boundaries of its available scanning field (46), and systematic optimization of acquisition parameters has been demonstrated to confer meaningful improvements in overall detection sensitivity for these peripherally distributed neovascular manifestations (47–49).

#### 4.1.4 Distinguishing NV from other retinal lesions

UWF-OCTA can effectively distinguish NV from similar lesions by virtue of depth-resolved imaging and ILM integrity display, which is a technical advantage over FA (28). Most signals on VRI Angio are NV, and a few are epiretinal membranes, venous loops, retinal tears or artifacts; these lesions can be distinguished from NV by B-scan and en face imaging (28, 50). IRMA is tortuous intraretinal vessels without ILM breakage, while NV protrudes into the vitreous cavity; UWF-OCTA can clearly distinguish the two, avoiding the interference of FA leakage on identification (33, 51, 52).

### 4.2 Non-perfusion areas (NPAs)

Capillary occlusion is widely regarded as a fundamental pathophysiological driver underlying the progression of DR through successive stages of severity (53–56). The presence and spatial extent of peripheral retinal nonperfusion have been causally associated with progressive visual deterioration and the development of measurable visual field impairment over time (57). Accordingly, systematic quantification of the degree and distribution of retinal ischemic burden has been proposed as a potentially valuable prognostic biomarker in DR, carrying meaningful implications for the individualization and optimization of patient-specific therapeutic decision-making algorithms (58, 59).

Employing UWF-FA as the reference standard, WF-OCTA has demonstrated commendable levels of both sensitivity and specificity in the detection of NPAs (44). Quantitative analyses utilizing 12 × 12 mm WF-OCTA acquisitions have yielded an Area Under the Curve (AUC) of 0.93 in discriminating eyes affected by DR of any severity from diabetic eyes without clinically evident retinopathy, alongside an AUC of 0.875 when differentiating non-proliferative DR (NPDR) eyes from diabetic individuals without retinopathic involvement (60). Given that capillary dropout in NPDR characteristically originates within the midperipheral retinal zone during the earliest disease stages, widefield imaging protocols are particularly well-positioned to furnish accurate and comprehensive DR severity grading. In a related investigation, Tan et al. (61) established that peripheral capillary dropout density (CCD) constitutes the most discriminating parameter for distinguishing mild NPDR from the absence of retinopathy, and further demonstrated that perfusion-related metrics derived from the retinal periphery possess substantially greater discriminative capacity for identifying advanced DR stages than measurements obtained from conventional small-field imaging formats.

WF-OCTA has clear limitations in NPA detection. It shows 98% sensitivity and 82% specificity for NPAs, with lower specificity than UWF-FA and potential false positives (44); it detects more NPAs than WF-FA due to refined microvascular dropout imaging, but the lack of deep capillary plexus (DCP)/superficial capillary plexus (SCP) separation may affect outcomes (58).

The narrower field of view of WF-OCTA restricts the capture of peripheral ischemic lesions (41, 42). Russell et al. (41) emphasized that WF-FA remains necessary to distinguish true nonperfusion from artifacts and Guo et al. (62) verified that over 50% of total NPAs are missed on 24 × 20 mm SS-OCTA compared with UWF-FA. WF-OCTA still accurately evaluates retinal ischemia within its scanning range.

Capillary occlusion promotes DR progression, and retinal ischemia assessment provides evidence for prognosis and treatment. WF-OCTA reliably quantifies central ischemia but misses most peripheral NPAs, while UWF-FA enables complete peripheral evaluation in an invasive manner. A tiered clinical strategy is feasible: WF-OCTA for initial NPDR/early PDR assessment, and UWF-FA when findings affect clinical decisions. The two modalities are complementary before the field of view of WF-OCTA is technically optimized.

### 4.3 Microaneurysms (MAs) and intraretinal microvascular abnormalities (IRMAs)

Microaneurysms (MAs) are recognized as among the earliest detectable clinical biomarkers heralding the onset of DR, while the systematic evaluation of IRMAs holds particular diagnostic significance as a critical determinant in differentiating severe NPDR from early proliferative DR (43, 63, 64). FA has long been established as the conventional reference standard for MA identification, given that these lesions characteristically manifest as discrete early hyperfluorescent foci accompanied by late-phase dye leakage on WF-FA imaging (63).

The capacity of WF-OCTA to reliably detect MAs may be attenuated by several inherent technical constraints, including the inability to resolve sluggish capillary flow falling below the instrument detection threshold, confounding projection artifacts, and signal attenuation within the deep capillary plexus (DCP) (65). Nonetheless, WF-OCTA uniquely affords depth-resolved anatomical localization of MA lesions within both the superficial capillary plexus (SCP) and DCP, thereby furnishing supplementary three-dimensional microvascular characterization that fundamentally transcends the capabilities of WF-FA (66). Taken together, these observations indicate that WF-OCTA and FA offer mutually complementary diagnostic advantages that, when integrated within a unified clinical assessment framework, yield a more comprehensive evaluation of MAs and IRMAs than either modality is capable of providing independently.

## 5 Emerging choroidal metrics in DR: exploratory evidence and current limitations

While DR has traditionally been considered primarily a retinal microvascular disease, there is growing recognition that the choroid may also be affected in diabetes. The choroid, which is the vascular layer located between the retina and the sclera, provides oxygen and nutrients to the outer retina, including the photoreceptors. However, in the context of the present review, it is important to emphasize that most available evidence regarding choroidal thickness, choroidal vascularity index (CVI), and choriocapillaris flow voids has been derived from standard OCT, enhanced depth imaging OCT, swept-source OCT, or conventional OCTA, rather than from true UWF-OCTA platforms. Therefore, these parameters should currently be regarded as exploratory complementary biomarkers rather than established UWF-OCTA-derived clinical indicators.

### 5.1 Choroidal thickness

Choroidal thickness can be measured using enhanced depth imaging OCT or SS-OCT, both of which provide improved visualization of the choroid compared to conventional spectral-domain OCT (67). Studies examining choroidal thickness in DR have yielded somewhat inconsistent findings, with some reporting thinning of the choroid in diabetic eyes and others reporting thickening or no significant difference (68, 69).

The variability in findings across studies may be attributable to several factors, including differences in the severity of DR among study populations, differences in the methods used to measure choroidal thickness (e.g., manual vs. automated segmentation, location of measurement), and the potential influence of confounding factors such as axial length, age, and blood pressure. It is also possible that choroidal thickness changes in a biphasic manner during the course of DR, with initial thickening due to inflammation or vascular congestion followed by later thinning due to choroidal degeneration and vascular dropout. Prospective longitudinal investigations of sufficient duration and methodological rigor are warranted to comprehensively elucidate the temporal trajectory and natural history of choroidal thickness alterations across the evolving continuum of diabetic ocular disease (see Table 1).

### 5.2 Choroidal vascularity index

The choroidal vascularity index (CVI), defined as the proportional relationship between the luminal cross-sectional area and the total choroidal area, has been advanced as a comparatively more stable and reproducible surrogate measure of choroidal integrity than choroidal thickness considered in isolation (68) reflects the relative composition of vascular and stromal compartments, CVI may be less susceptible to certain physiological confounders that influence absolute thickness measurements. Several studies have reported reduced CVI in diabetic eyes, particularly in more advanced stages of DR, suggesting possible choroidal vascular compromise (70). This finding could be interpreted as evidence of choroidal vascular degeneration in diabetes, which might contribute to outer retinal ischemia and photoreceptor dysfunction. Nevertheless, the evidence remains heterogeneous, and not all studies have demonstrated consistent differences between diabetic and non-diabetic eyes or across DR severity groups. At present, the relationship between CVI, DR progression, treatment response, and prognosis remains insufficiently established, and its clinical utility as an actionable biomarker requires further validation in larger, longitudinal, and methodologically standardized cohorts (71).

### 5.3 Choroidal flow voids on OCTA

OCTA-based assessment of the choriocapillaris has also introduced the concept of flow voids, which refer to areas of reduced or absent flow signal on en face choriocapillaris images (69). These flow voids are thought to represent areas of choriocapillaris dropout or nonperfusion, as schematically summarized in Supplementary Figure 1. Increased number or size of choriocapillaris flow voids has been described in diabetic eyes and has been reported to correlate with DR severity and the presence of diabetic macular edema in some cohorts (72). However, interpretation of these findings requires caution. Choriocapillaris imaging is highly sensitive to segmentation errors, signal attenuation, projection artifacts from overlying retinal vessels, and differences in image-processing thresholds. Moreover, available studies have not yet established whether these flow deficits represent independent predictors of disease progression, treatment response, or visual prognosis.

**Figure 3:**
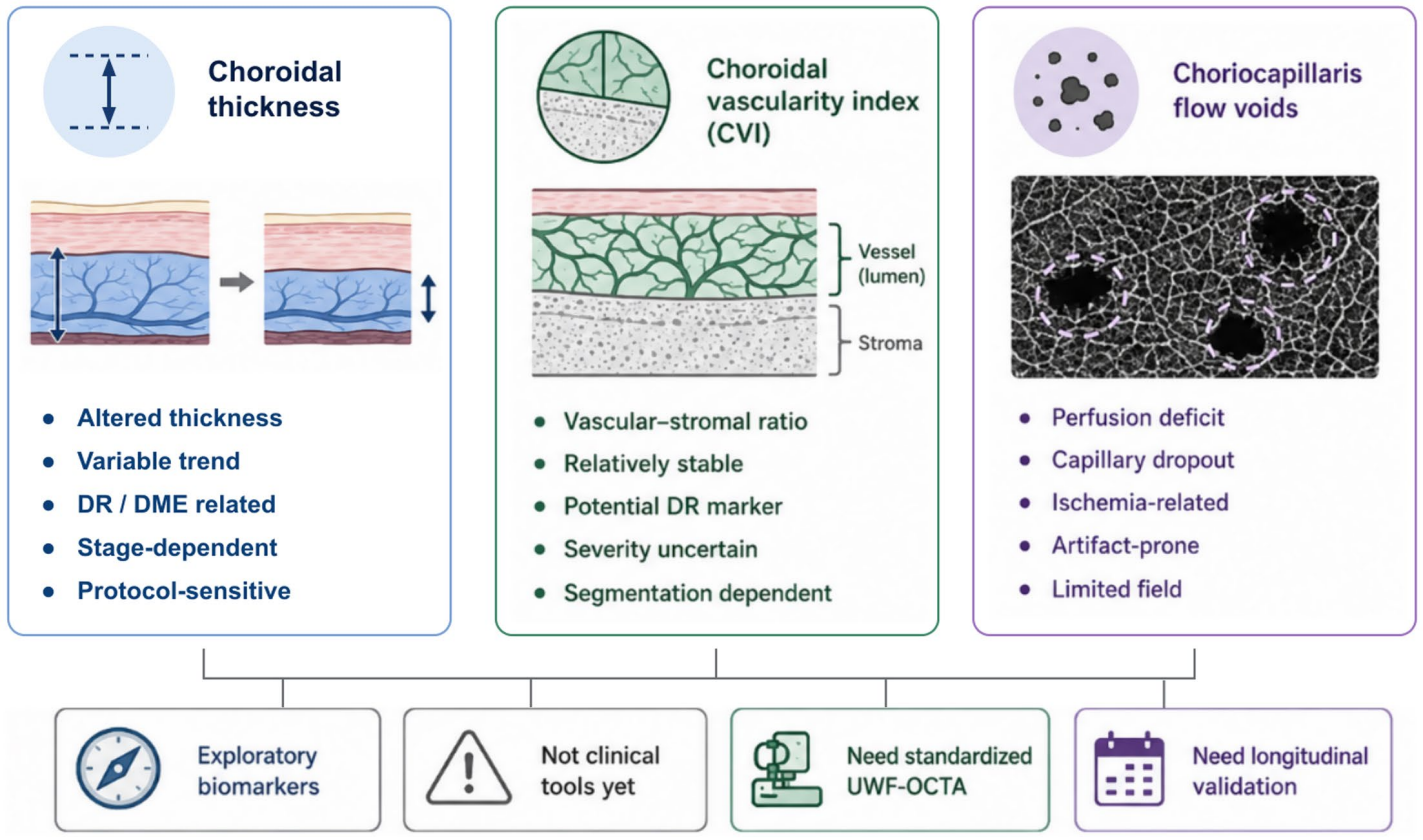
Illustrative summary of choroidal biomarker patterns across the spectrum of diabetic retinopathy. This schematic summarizes reported patterns and current limitations regarding choroidal changes across the spectrum of DR. Choroidal thickness has been variably reported across studies, with some evidence suggesting possible thickening in diabetic eyes without clinically visible DR and thinning in more advanced stages; however, this pattern remains insufficiently validated and should be interpreted as exploratory rather than progressive. CVI has been reported to decrease in some studies with increasing DR severity, potentially reflecting changes in the relative proportions of stromal and vascular components, although findings remain heterogeneous. Choriocapillaris flow voids may increase in number and size in diabetic eyes and have been reported to correlate with diabetic macular edema and retinal NPAs in some cohorts. Overall, this figure is intended as a hypothesis-generating visual summary of current evidence and methodological limitations, not as an empirically validated model of choroidal disease progression. DR, diabetic retinopathy; NPDR, non-proliferative diabetic retinopathy; PDR, proliferative DR; CVI, choroidal vascularity index; NPAs, non-perfusion areas.

Taken together, choroidal metrics may provide useful mechanistic information regarding diabetic ocular microvascular involvement, but their relevance to UWF-OCTA-based DR assessment remains preliminary. Current evidence is limited by the predominance of non-UWF imaging sources, heterogeneity of acquisition and analysis protocols, and inconsistent associations with DR severity and clinical outcomes. Accordingly, Figure 3 is presented as an illustrative, hypothesis-generating summary of reported choroidal biomarker patterns, current evidence, methodological limitations, and future research directions in DR, rather than as an empirically validated model of progressive choroidal pathology. Further prospective studies using standardized UWF-OCTA protocols, consistent segmentation strategies, and longitudinal clinical endpoints are needed before choroidal parameters can be incorporated into routine DR risk stratification or treatment monitoring.

## 6 Comparison between UWF-OCTA and FA

The evidence reviewed above indicates that UWF-OCTA presents favorable performance in the detection of typical retinal lesions and the evaluation of choroidal metrics in DR, especially showing unique advantages in identifying core lesions associated with PDR. However, the clinical application value of UWF-OCTA still needs to be further clarified by comprehensive comparison with FA, the traditional reference standard in DR imaging. By systematically analyzing the differences between UWF-OCTA and FA in lesion detection capability and clinical functional assessment, it can be concluded that the two modalities are not mutually substitutive but play a complementary role in the clinical diagnosis and management of DR.

The comparison between UWF-OCTA and UWF-FA has been the subject of several studies, and the findings to date suggest that these two modalities have complementary strengths and weaknesses rather than one being uniformly superior to the other. To present this comparison in a more clinically relevant and logically coherent manner, the following discussion is organized into two distinct dimensions: (1) lesion detection capability, focusing on the identification of specific pathological features such as NV, IRMAs, NPAs, and MAs; and (2) clinical functional assessment, including leakage evaluation, disease activity monitoring, and feasibility for longitudinal follow-up (Table 2).

**Table 2:** Comparative advantages and limitations of UWF-OCTA and FA in DR.

Feature	UWF-OCTA	UWF-FA
Invasiveness	Non-invasive; no dye	Invasive; intravenous dye
Acquisition time	Seconds per eye	Several minutes per eye
Field of view (single capture)	40°–60° single-shot; broader with montage	Up to about 200° on UWF systems
Depth resolution	Yes; retinal plexuses; selected choriocapillaris assessment	No; two-dimensional projection
Quantitative metrics	Yes: VD, PD, FAZ, NV area; exploratory choroidal metrics	Limited; qualitative or semi-quantitative
Leakage assessment	No	Yes; reference for activity
Differentiation of NV from IRMA	Yes (based on ILM breach)	Limited; leakage confounds
MA detection	May miss slow-flow MAs	High sensitivity
Risk of allergy/nephrotoxicity	None	Small but non-negligible
Segmentation error susceptibility	High (ILM, retinal layers)	Low
Motion artifact susceptibility	Moderate to high	Low
Peripheral NPA detection	May miss far-peripheral NPAs	More comprehensive peripheral coverage

Before addressing these comparisons in detail, however, an important clarification regarding the reference standard is warranted. FA has long been accorded the status of the reference gold standard modality for the comprehensive evaluation of vascular leakage dynamics and blood-retinal barrier integrity, parameters that collectively constitute indispensable determinants in establishing patient eligibility for therapeutic intervention and in objectively quantifying disease activity across the clinical spectrum of DR. From a clinical perspective, the presence and extent of leakage on FA remains the most direct indicator of active NV and diabetic macular edema. Nevertheless, it is worth noting that FA does not serve as an absolute pathological gold standard for all aspects of lesion characterization. For morphological assessments, particularly the precise structural delineation of NV, differentiation of NV from IRMA, and visualization of intraretinal vascular details, FA has inherent limitations due to its two-dimensional projection nature and the confounding effect of dye leakage, which often obscures the underlying vascular architecture. In these specific contexts, OCTA, especially when combined with structural B-scan correlation, offers unique and, in some respects, superior information.

### 6.1 Lesion detection capability

#### 6.1.1 NV and differentiation from IRMA

**Figure 4:**
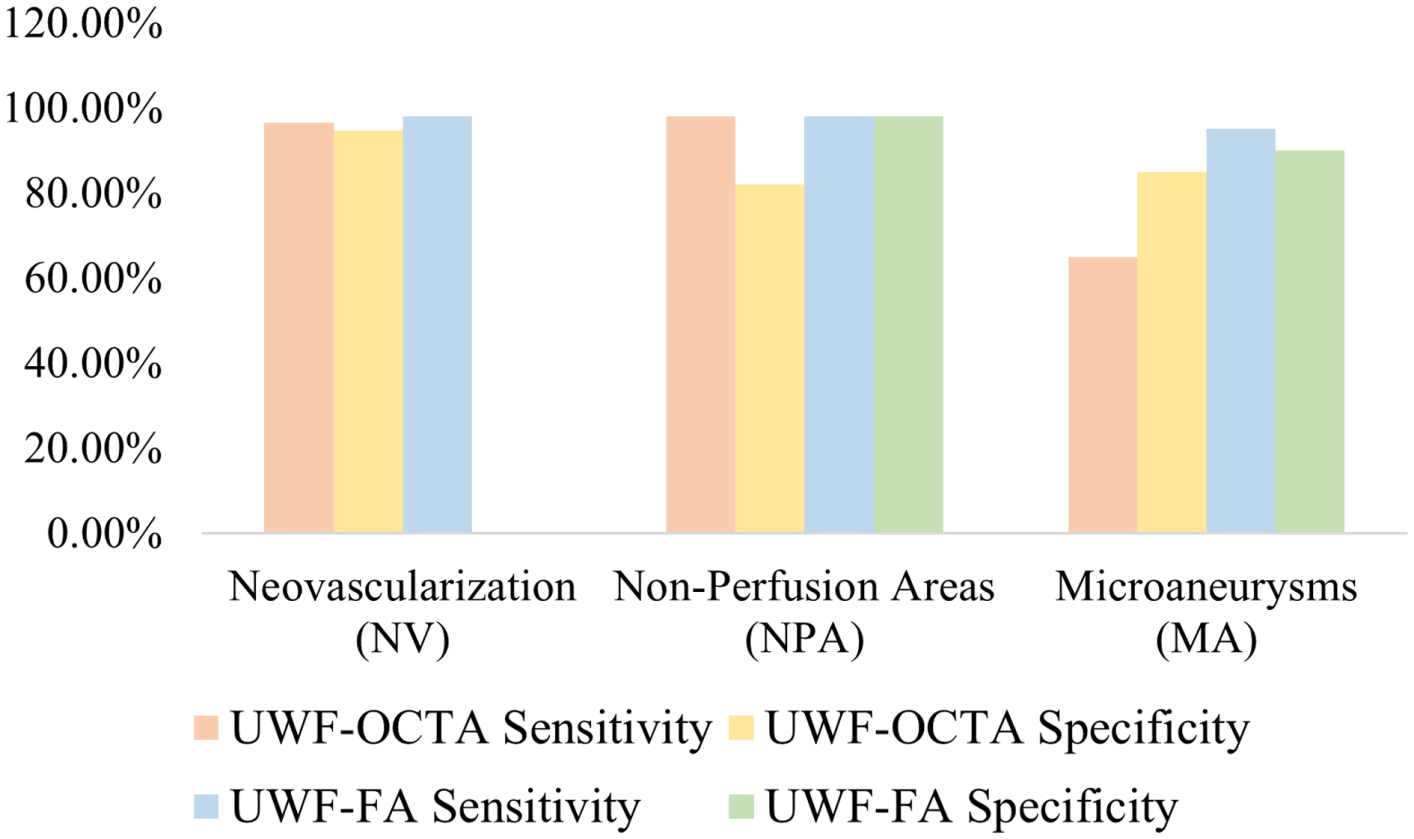
Diagnostic performance of UWF-OCTA compared to FA for detecting DR lesions. Data compiled from Pourbagherkhah and Jaldian (45), Russell et al. (41–43), Sawada et al. (44). FA serves as the reference standard for NV and NPA detection; thus, sensitivity and specificity values for FA are not directly comparable in the same manner as for OCTA. MA detection by OCTA is limited by slow flow below the detection threshold.

In the detection of neovascularization (NV) which is the core lesion of proliferative diabetic retinopathy (PDR), UWF-OCTA shows high sensitivity and specificity that are generally comparable to UWF-FA in a certain degree (Figure 4) (45). The detection efficiency of UWF-OCTA tends to be affected by NV morphological types to some extent. Flat NV limited to the posterior hyaloid face without breaking through the ILM are hard to identify on automated VRI Angio slabs due to segmentation errors, with the detection rate as low as 67.9% in related studies (28), while forward and tabletop NV extending into the vitreous cavity can be detected with considerable ideal accuracy (98.6–100%).

More importantly, UWF-OCTA has obvious advantages in differentiating NV from IRMA for PDR staging. On FA images, NV and IRMA both show hyperfluorescence with late leakage, bringing difficulties to accurate identification. In contrast, UWF-OCTA can clearly display the structural difference that NV breaks through the ILM and stretches into the vitreous while IRMA is confined in retinal layers, and this advantage may have important clinical value for precise PDR severity grading and treatment guidance (52).

#### 6.1.2 Non-perfusion areas (NPAs)

For PDR-related peripheral ischemia assessment, UWF-OCTA has high sensitivity but relatively low specificity in NPA detection compared with UWF-FA (Figure 4) (44), which may be related to false-positive flow voids caused by poor signal quality and segmentation errors. The limited field of view of UWF-OCTA may affect the comprehensive evaluation of peripheral NPAs that are closely associated with PDR progression. Research shows that more than 50% of peripheral NPAs can be missed on 24 × 20 mm SS-OCTA scans relative to UWF-FA (62), though UWF-OCTA can still accurately reflect the ischemia degree within its scanning range.

#### 6.1.3 MAs and IRMAs

MA is a typical lesion of non-PDR DR, and UWF-FA is still superior in slow-flow MA detection (63), while UWF-OCTA is prone to miss MAs due to low flow velocity and artifacts (65).

For PDR clinical judgment, the core value of UWF-OCTA lies in distinguishing IRMA from NV, and its depth-resolved localization ability of microvasculature can provide supplementary information for differential diagnosis (66), which helps to reduce the misjudgment between severe NPDR and early PDR. The diagnostic performance of UWF-OCTA and UWF-FA in detecting typical DR lesions is compared in Figure 4.

### 6.2 Clinical functional assessment

#### 6.2.1 Leakage assessment and disease activity

UWF-FA remains the gold standard for assessing vascular leakage, which is a critical parameter for determining disease activity in DR. The presence and pattern of leakage directly inform clinical decisions regarding anti-VEGF therapy, as active NV is typically characterized by progressive hyper fluorescence on late-phase FA images.

UWF-OCTA does not provide information about vascular leakage. Although the morphological characteristics of NV as depicted on OCTA imaging may furnish certain inferential indicators regarding lesion activity status, with actively proliferating NV typically manifesting as an exuberant and disorganized arborization of fine-caliber vessels in contrast to the pruned, filamentous vascular loops that characterize quiescent or regressed NV, such morphology-based activity assessment is by its very nature an indirect evaluative approach that remains inherently susceptible to inter-observer variability and a degree of subjectivity that precludes definitive conclusions regarding lesion activity in individual clinical encounters (71). Therefore, for treatment-naïve patients or those with suspected disease reactivation, FA continues to play an essential role.

#### 6.2.2 Follow-up feasibility and monitoring

For patients requiring frequent monitoring (e.g., those receiving anti-VEGF therapy), UWF-OCTA offers several practical advantages. It is non-invasive, can be performed rapidly (typically within seconds per eye), and provides quantitative metrics (vessel density (VD), perfusion density (PD), NV area, foveal avascular zone (FAZ) parameters) that are more objective and reproducible than the qualitative assessment of leakage on FA. These quantitative features make UWF-OCTA a potentially useful tool for tracking disease progression or regression over time.

However, longitudinal assessment with UWF-OCTA is not without challenges. Segmentation errors may vary from visit to visit, introducing measurement variability. Motion artifacts and signal attenuation in eyes with media opacities can also compromise image quality. Nonetheless, with careful quality control and, when necessary, manual segmentation correction, UWF-OCTA appears to be a feasible option for serial monitoring (34).

### 6.3 Integrated comparison and clinical implications

Table 2 provides a side-by-side comparison of the two modalities across key technical and clinical parameters. Viewed in their totality, the cumulative findings drawn from the available body of evidence converge upon the conclusion that UWF-OCTA and UWF-FA are most appropriately conceptualized as mutually reinforcing diagnostic instruments that operate in a synergistic and complementary capacity, rather than representing competing or interchangeable modalities between which a definitive clinical choice must be made.

In clinical practice, a pragmatic approach might involve the following: UWF-OCTA could serve as a great screening tool for PDR, given its non-invasive nature and high sensitivity for NV detection. Patients with suspected NV or those requiring leakage assessment for treatment decisions could then undergo FA for confirmation and activity evaluation. For patients requiring frequent monitoring, such as those on anti-VEGF therapy, UWF-OCTA may be preferred due to its safety and quantitative capabilities. For patients with extensive peripheral disease or suboptimal image quality on OCTA, FA remains necessary. Ultimately, the choice of imaging modality should be guided by the specific clinical question being asked, local availability and expertise, and individual patient characteristics.

### 6.4 Limitations in the available comparative evidence

Acknowledging the limitations of the current literature is important. Most comparative studies have relatively small sample sizes, use different UWF-OCTA devices and acquisition protocols, and employ varying criteria for lesion identification. This heterogeneity makes it difficult to draw definitive, universally applicable conclusions. A further methodological limitation of considerable significance lies in the predominately cross-sectional nature of the existing comparative literature, as prospective longitudinal datasets specifically designed to evaluate and contrast the performance of these two modalities in the context of therapeutic response monitoring remain conspicuously scarce. Addressing this evidence gap will necessitate the undertaking of rigorously designed, large-scale, prospective investigations conducted across multiple participating centers and governed by uniformly standardized imaging acquisition and analysis protocols, efforts that are essential to more precisely delineating and formally establishing the respective and complementary clinical roles of UWF-OCTA and UWF-FA within the evolving landscape of DR diagnosis, staging, and long-term management.

### 6.5 Clinical application boundaries: practical clinical considerations

Based on the comparative evidence summarized above, the following statements should be interpreted as practical clinical considerations rather than formal clinical guidelines or consensus recommendations. These considerations are derived from the current literature and have not undergone standardized evidence grading or formal consensus development. Across available studies, the evidence generally supports a complementary relationship between UWF-OCTA and FA: UWF-OCTA provides non-invasive, depth-resolved, and quantitatively analyzable vascular information, whereas FA remains necessary when leakage activity, far-peripheral ischemia, or dynamic dye-based assessment is clinically required.

In selected clinical contexts, UWF-OCTA may be considered as an initial imaging modality, particularly for patients requiring repeated follow-up, those with contraindications or intolerance to fluorescein dye, and patients in whom non-invasive evaluation of NV or progression from NPDR toward PDR is clinically relevant. Its potential value lies in repeated structural assessment, quantitative monitoring of vascular changes, and differentiation between NV and IRMA through depth-resolved evaluation of ILM breach. However, these applications remain dependent on image quality, scan coverage, segmentation accuracy, and local technical expertise.

Supplemental FA should be considered when OCTA findings are negative, equivocal, or discordant with clinical examination; when flat, far-peripheral, or slow-flow NV is suspected; when vascular leakage or lesion activity must be assessed before treatment; or when the extent of peripheral non-perfusion may influence management. FA also remains important in active PDR, diabetic macular edema, preoperative assessment of advanced PDR, and research settings in which leakage is a primary endpoint.

Several factors currently limit broader substitution of FA by UWF-OCTA, including the lack of standardized acquisition protocols, incomplete detection of flat or peripheral NV, inability to directly assess leakage, segmentation variability, limited longitudinal outcome data, and insufficient cost-effectiveness evidence. Further multicenter studies with standardized imaging protocols and clinically relevant endpoints are required before these clinical considerations can be translated into formal recommendations.

## 7 Future directions

Several future research areas for UWF-OCTA in DR require attention. Although UWF-OCTA has shown certain application value in DR assessment, current research and clinical practice still have numerous unresolved limitations, such as non-unified imaging specifications, insufficient automatic lesion recognition accuracy, and unclear guidance value of quantitative indicators. In view of these practical bottlenecks, subsequent research of UWF-OCTA in DR can be promoted step by step in combination with short-term, medium-term and long-term layouts, so as to gradually improve its clinical application system.

In the short term, deep learning can be prioritized to optimize the automatic lesion detection capability of UWF-OCTA. Flat NV is often difficult to identify accurately due to ILM segmentation limitations, peripheral scan images have relatively low signal-to-noise ratio, and severe DME will also disturb the integrity of retinal structure to a certain extent. Constructing deep learning models with diverse training data covering the above complex scenarios and conducting rigorous cross-device and cross-center verification will help to steadily improve the accuracy of automatic identification of key lesions.

In the medium term, large-scale longitudinal and horizontal comparative studies should be carried out simultaneously to clarify the clinical value of UWF-OCTA-related indicators. Long-term follow-up of patients during the transition from NPDR to PDR and treatment can help explore the temporal correlation between choroidal and retinal vascular changes, and determine the predictive effect of relevant indicators on clinical outcomes. Meanwhile, prospective comparative studies between UWF-OCTA-based evaluation algorithms and FA or clinical examination are needed to confirm whether quantitative indicators such as NV area and vessel density can guide the formulation of clinical strategies including anti-VEGF treatment timing and panretinal photocoagulation (PRP). In addition, further exploration of choroidal metrics is also an important part of medium-term research, including clarifying the sequence of choroidal thinning and retinal damage, verifying the response of choroidal parameters to anti-VEGF therapy, and standardizing the quantification method of choroidal flow voids, so as to establish choroidal metrics as effective clinical biomarkers.

In the long run, establishing multi-center consensus and a standardized UWF-OCTA imaging system is the core task. Current inconsistencies in devices, scan protocols, segmentation algorithms and analysis methods limit the generalizability of research results. Developing unified standards for imaging acquisition, reporting and quality control through multi-center cooperation will lay the foundation for cross-study data integration and promote the standardized application of UWF-OCTA in DR clinical management.

## 8 Conclusion

UWF-OCTA constitutes a pivotal technical advancement in diabetic retinopathy imaging, overcoming the field-of-view constraints of conventional OCTA while retaining non-invasiveness and high-resolution depth-resolved visualization. It enables reliable detection of key retinal microvascular lesions—especially neovascularization via the vitreoretinal interface slab—and delivers quantitative metrics for longitudinal disease monitoring, with emerging choroidal indicators further enriching the multidimensional assessment of DR pathophysiology. Critically, UWF-OCTA serves as a valuable complementary structural imaging tool rather than a replacement for fluorescein angiography: it excels in non-invasive lesion identification and quantitative follow-up, yet FA remains irreplaceable for evaluating vascular leakage and comprehensive peripheral retinal ischemia, which are essential for guiding therapeutic decisions in active DR. With future standardization of imaging protocols and validation of quantitative biomarkers, UWF-OCTA is poised to optimize DR screening, risk stratification, and treatment monitoring workflows, advancing personalized management of diabetic retinal vascular disease.

## Glossary

AUC: Area Under the Curve
CVI: Choroidal Vascularity Index
CCD: Capillary Dropout Density
DCP: Deep Capillary Plexus
DME: Diabetic Macular Edema
DR: Diabetic Retinopathy
DRSS: Diabetic Retinopathy Severity Scale
EFI: Extended Field Imaging
ETDRS: Early Treatment Diabetic Retinopathy Study
FA: Fluorescein Angiography
FAZ: Foveal Avascular Zone
ILM: Internal Limiting Membrane
IRMAs: Intraretinal Microvascular Abnormalities
MAs: Microaneurysms
NPAs: Non-Perfusion Areas
NPDR: Non-Proliferative Diabetic Retinopathy
NV: Neovascularization
NVD: Neovascularization of the Disc
NVE: Neovascularization Elsewhere
NPV: Negative Predictive Value
OCT: Optical Coherence Tomography
OCTA: Optical Coherence Tomography Angiography
PD: Perfusion Density
PRP: Panretinal Photocoagulation
PDR: Proliferative Diabetic Retinopathy
RPE: Retinal Pigment Epithelium
SCP: Superficial Capillary Plexus
SS-OCTA: Swept-Source Optical Coherence Tomography Angiography
UWF-FA: Ultra-Widefield Fluorescein Angiography
UWF-OCTA: Ultra-Widefield Optical Coherence Tomography Angiography
VRI: Vitreoretinal Interface
VD: Vessel Density
