# Supplementary figures and images for: Ultra-widefield optical coherence tomography angiography in diabetic retinopathy: from retinal lesions to choroidal metrics
Source: Front Med (Lausanne). 2026 Jun 22;13:1885710. doi: 10.3389/fmed.2026.1885710 (PMC13333442; doi:10.3389/fmed.2026.1885710)

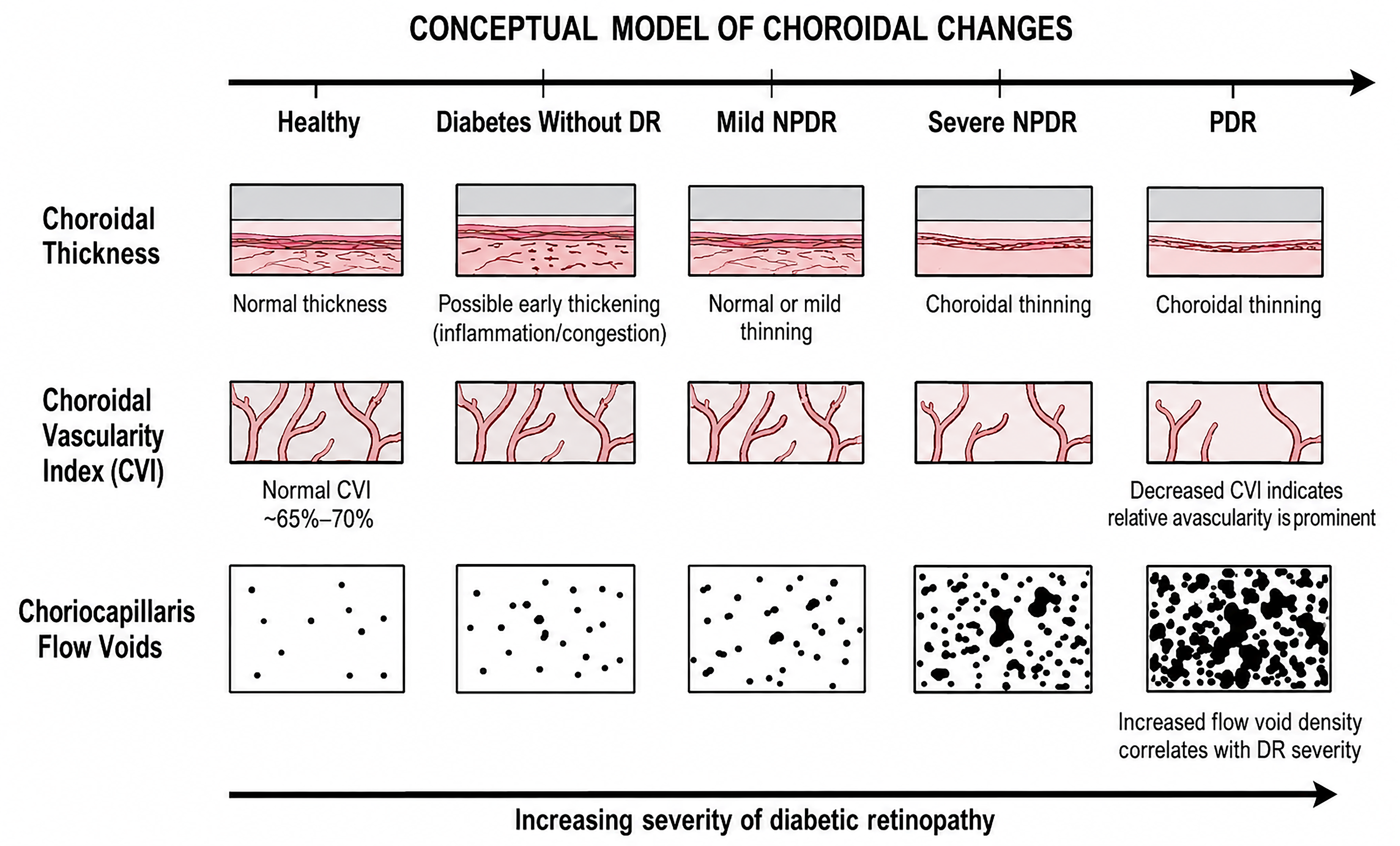

Supplement: Supplementary file 1 [file Image_1.TIF]
